# Childhood trauma and physical activity link immunometabolic biomarkers and psychiatric symptoms in medically healthy adults

**DOI:** 10.1017/neu.2026.10069

**Published:** 2026-03-25

**Authors:** Gemma Wallace, Quincy Beck, Leslie Brick, Teresa Daniels, Asi Gobin, Stephanie Parade, Audrey Tyrka

**Affiliations:** 1 Department of Psychiatry and Human Behavior, Brown University, USA; 2 Psychosocial Research Program, Butler Hospital, USA; 3 Mood Disorders Research Program and Laboratory for Clinical and Translational Neuroscience, Butler Hospital, USA; 4 Center on Alcohol, Substance Use, and Addiction, University of New Mexico, USA; 5 Bradley/Hasbro Children’s Research Center, Bradley Hospital, USA

**Keywords:** psychopathology, insulin resistance, inflammation, adverse childhood experiences, health behavior

## Abstract

**Objective:**

The comorbidity of psychiatric and metabolic conditions is prevalent and poses a heavy burden on public health. Several biopsychosocial factors are known to influence both metabolic and psychiatric health, including inflammation, eating behavior, physical activity, and early life stress. Few studies, however, have examined the constellation of interrelationships among multiple risk domains simultaneously.

**Methods:**

Using a sample of 200 medically healthy adults enrolled in a parent study, we used Gaussian Graphical Modeling, a type of network analysis, to characterize interdependent cross-sectional associations between early life stress (childhood trauma), health behaviors (diet quality and physical activity), blood-based biomarkers of metabolic functioning (insulin resistance, HDL cholesterol, triglycerides) and inflammation (C-reactive protein [CRP]), and three domains of mental health symptoms (depressive, anxious, and post-traumatic stress symptoms). We hypothesized that the network structure would highlight a pattern whereby higher CRP, poorer diet quality, lower physical activity, and higher childhood trauma would associate with increased risk for both metabolic and psychiatric impairments.

**Results:**

Findings revealed a positive conditional association between CRP and childhood trauma, which may function as an intermediary process to increase risk for both metabolic impairments and psychiatric symptoms in adulthood. Further, higher physical activity was associated with lower insulin resistance and fewer depressive symptoms, and better diet quality was associated with lower CRP levels.

**Conclusion:**

Results highlight potential avenues for interventions aimed at reducing inflammation, improving health behavior, and addressing the effects of childhood trauma to improve physical and mental health comorbidities.

Significant outcomes

- Insulin resistance had direct and potential indirect associations with other immunometabolic biomarkers and psychiatric symptoms. Thus, glycemic dysregulation may represent a novel intervention target for improving psychiatric health.
- Early life stress may have pro-inflammatory effects that increase risk for both metabolic and psychiatric problems in adulthood.
- Health behaviors, including physical activity and diet quality, may protect against both immunometabolic and psychiatric problems in at-risk individuals.

Limitations

- The cross-sectional design prevented interpreting causal or temporal effects. Employing longitudinal approaches could clarify the timescales and causal directions of observed associations.
- Our sample was 68% White and 83% non-Hispanic, and it is important to replicate results in more diverse samples.
- It would be valuable to consider additional relevant physiological biomarkers and socioenvironmental contexts that influence both psychiatric and metabolic health.

## Introduction

The co-occurrence of psychiatric and physical health conditions is increasingly recognized as a major contributor to the global public health burden (Momen *et al*., 2020). Psychiatric and metabolic conditions, such as type 2 diabetes, metabolic syndrome, and hyperlipidemia, are particularly prevalent and highly comorbid. Psychiatric patients frequently have indicators of metabolic dysfunction, such as impaired glucose metabolism (Koponen *et al*., 2015; Steardo *et al*., 2019), abnormal blood lipid profiles (e.g., elevated triglycerides and reduced high-density lipoprotein [HDL] cholesterol) (Wysokiński *et al*., 2015; Li *et al*., 2023), and obesity (Afzal *et al*., 2021). While over 40% of adults in the United States are affected by metabolic syndrome (Liang *et al*., 2023), psychiatric patients experience up to 58% higher rates of metabolic syndrome compared to the general population (Vancampfort *et al*., 2015; Penninx & Lange, 2018). Further, a growing body of evidence suggests these associations are bidirectional. Indicators of metabolic dysfunction are prospective risk factors for developing psychiatric conditions (Perry *et al*., 2021; Chourpiliadis *et al*., 2024), and conversely, psychiatric conditions increase risk for the subsequent onset of metabolic problems (Mezuk *et al*., 2008). Despite the robust literature on reciprocal relations between psychiatric and metabolic problems, the processes underlying these associations remain unclear. Understanding potential pathways that link psychiatric and metabolic problems may identify opportunities for critically needed intervention and prevention strategies.

Several interrelated biopsychosocial factors influence both metabolic and psychiatric health, and the comorbidity of these conditions is likely multifaceted and complex. Elevated pro-inflammatory immune system activity is increasingly recognized as a transdiagnostic feature of several psychiatric symptom profiles, including depression (Miller & Raison, 2016), anxiety (Peirce & Alviña, 2019), posttraumatic stress disorder (Hori & Kim, 2019), and other psychiatric diagnoses (Miller, 2020). Inflammation may contribute to psychiatric symptoms via dysregulation of neurotransmitter, neurocircuitry, and stress response systems, which can impair affective and cognitive functioning (Brundin *et al*., 2017; Berardelli *et al*., 2020). Further, inflammation has reciprocal associations with metabolic dysfunction, referred to as immunometabolism, in which elevated inflammation and metabolic problems can exacerbate and perpetuate each other (Hotamisligil, 2006; Makowski *et al*., 2020; Fahed *et al*., 2022). C-reactive protein (CRP) is an acute-phase reactant produced in the liver and a well-established biomarker of inflammation. CRP levels are associated with metabolic syndrome and cardiovascular disease (Devaraj *et al*., 2009), as well as childhood trauma and several health behaviors such as physical activity and eating behaviors (Schrepf *et al*., 2014; You, 2024). In turn, many health behaviors, particularly diet and physical activity also significantly impact both psychiatric and metabolic health. Poor diet quality and physical inactivity increase risk for both psychiatric and metabolic problems (Zinöcker & Lindseth, 2018; Bremner *et al*., 2020; Firth, Gangwisch, *et al*., 2020; Firth, Solmi, *et al.*, 2020; Thyfault & Bergouignan, 2020), and these effects are likely influenced by inflammatory pathways (Hotamisligil, 2006; Kim *et al*., 2022; Yuguang *et al*., 2024). The role of diet and physical activity is particularly important to explore given their potential as modifiable targets for behavioral intervention.

Early life stress, including childhood trauma, abuse, and neglect, may also be a key player in metabolic and psychiatric comorbidities (Gómez-Ilescas & Silveira, 2025b). It is well-established that early life stress increases vulnerability to poorer metabolic and psychiatric outcomes and higher mortality in adulthood (Kessler *et al*., 2010; Merrick *et al*., 2019; Deschênes *et al*., 2025). This may be related to the long-term impact of early life stress on multiple physiological and psychological systems including, but not limited to, elevated inflammation (Fagundes & Way, 2014), increased stress sensitivity (McLaughlin *et al*., 2010), and less favorable health behaviors in adulthood (Gavrieli *et al*., 2015; Duffy *et al*., 2018). Taken together, psychiatric and metabolic comorbidities are likely impacted by interrelations among inflammation, health behaviors, and early life stress. However, to our knowledge, potential pathways among these constructs have rarely been examined simultaneously, and most prior studies have focused on only one domain of mental health symptoms (e.g., posttraumatic stress or depressive symptoms only) (Matta *et al*., 2019; Lyu *et al*., 2024). The transdiagnostic nature of metabolic and psychiatric comorbidities warrants examining these processes concurrently across multiple domains of mental health symptoms.

The current study aimed to characterize interdependent associations between early life stress (childhood trauma), health behaviors (diet quality and physical activity), blood-based biomarkers of metabolic functioning (insulin resistance, HDL cholesterol, triglycerides) and inflammation (C-reactive protein [CRP]), and three domains of mental health symptoms (depressive, anxious, and post-traumatic stress symptoms). We employed a cross-sectional network analysis to identify the interrelations between these constructs in a community sample of medically healthy adults with and without early life stress. Network models estimate and visualize associations between each pair of variables while adjusting for other paths in the model and thus may uncover direct and potential indirect pathways between sets of variables (Borsboom *et al*., 2021). We hypothesized that the network structure would identify potential pathways between metabolic and mental health variables, in which higher inflammation (i.e., higher CRP levels), poorer diet quality, lower physical activity, and higher early life stress, as well as interrelations between these constructs, would correlate with increased risk for both metabolic and psychiatric impairments.

## Material and methods

### Participants and procedures

The current study used secondary data from the Lifestyle Influences of Family Environment (LIFE) study, which investigated relationships of chronic childhood adversity on epigenetics, neuroendocrine and immune function, and behavioral health. Participants were recruited via advertisements that were posted on community boards, social media websites, and a hospital website seeking medically healthy adults who experienced early life stress, including childhood maltreatment and parental loss, as well as adults from two-parent households without early life stress. Prior to enrollment, a brief phone call was used to screen potential participants to assess whether study eligibility criteria were met, including review of inclusion and exclusion criteria. Participants were informed of the voluntary nature of the study and written informed consent was obtained. Inclusion criteria included being 18–40 years old and, for the early life stress group, moderate–severe maltreatment prior to age 18 as defined by the Childhood Experience of Care and Abuse interview (CECA) (Bifulco *et al*., 1994; Bifulco *et al*., 1997). The early life stress group was enriched for individuals who experienced parental loss before age 18 (*n* = 92), and participants in the control group had no major history of psychiatric disorders or childhood maltreatment and were raised in two-parent homes with no history of parental separation, divorce, or loss. Exclusion criteria included acute and chronic medical conditions, pregnancy, and medications other than hormonal contraceptives, due to the potential for these factors to impact study analytes. Additionally, due to confounding genetic and physiological processes associated with primary bipolar disorder, obsessive–compulsive disorder (OCD), and psychotic disorders, these conditions were also exclusionary. Psychiatric diagnoses were confirmed via the Mini-International Neuropsychiatric Interview (Sheehan *et al*., 1998) adapted for the DSM-5 (*n* = 35) or the Structured Clinical Interview for DSM-5 (First *et al*., 2015) (*n* = 165). Current drug use (confirmed by positive drug screens) and current substance use disorders were initially exclusionary. However, as cannabis use became more prevalent in community samples during the study years (mid-to-late 2010s), we allowed participants who self-reported cannabis use (*n* = 59) to enroll during later phases of recruitment to maintain recruitment feasibility. Participants who reported current heavy cannabis use (defined as use ≥3 times per week) were excluded throughout the study. Refer to Daniels *et al*. (2023) for additional details on the LIFE study sample and procedures (Daniels *et al*., 2023).

Once enrolled, participants completed baseline in-person fasting blood draws, clinical interviews, and several self-report assessments of early adversity, health, and behavior. Except where indicated, all data for the current study came from the baseline visit, as most variables of interest were not measured at subsequent follow-up visits. While the primary LIFE study enrolled a total of 220 participants, 13 participants were excluded due to not providing blood samples or sufficient data for study group determination, four participants withdrew after enrollment, and 3 participants had questionable veracity of report. Thus, the analytic sample for the current study included *N* = 200 adults, including *n* = 118 and *n* = 82 with and without early-life stress, respectively. Self-reported demographic characteristics for the analytic sample are shown in Table 1. Participants provided informed consent for their participation, and all study procedures were approved by the Institutional Review Board of Butler Hospital (IRB Protocol #793444).

**Table 1.** Self-reported demographic characteristics of the analytic sample (*N* = 200)

Characteristic	Mean (*SD*) or *n* (%)
Age (in years)	27.28 (5.66)
Sex assigned at birth	
Female	137 (68.5%)
Male	63 (31.5%)
Gender	
Man	46 (23.0%)
Woman	93 (46.5%)
Other	5 (2.5%)
Unknown or not reported	56 (28.0%)
Ethnicity	
Hispanic or Latine	34 (17.0%)
Not Hispanic or Latine	166 (83.0%)
Race	
American Indian or Alaska Native	4 (2.0%)
Asian	13 (6.5%)
Black or African American	20 (10.0%)
Multiracial	18 (9.0%)
White or Caucasian	136 (68.0%)
Unknown or not reported	9 (4.5%)
Highest level of education	
Less than 7 years of school	1 (0.5%)
Junior high school	1 (0.5%)
Partial high school	5 (2.5%)
High school graduate	17 (8.5%)
Technical school	6 (3.0%)
Partial college	62 (31.0%)
College graduate	86 (43.0%)
Professional degree	22 (11.0%)
Annual household income	
$0–$24 999	42 (21.0%)
$25 000–$49 999	55 (27.5%)
$50 000–$74 999	31 (15.5%)
$75 000–$99 999	22 (11.0%)
$100 000+	28 (14.0%)
Unknown or not reported	22 (11.0%)

### Measures

#### Immunometabolic biomarkers

*Blood sample collection.* Plasma samples for biospecimen assays were obtained via fasting blood draws. Participants were instructed to fast (with the exception of water) starting at 8:00 PM the night prior, and venipuncture was performed between 8:15–9:00 AM the following day. Blood samples were drawn from the antecubital region by a trained phlebotomist or research nurse using 10 mL EDTA plasma tubes and 23- to 21-guage butterfly needles. For most biomarkers, samples remained uncentrifuged and were sent immediately to the laboratory for analysis; if samples needed to be stored overnight before analysis, they were stored at approximately 40 °F and centrifuged prior to being assayed the following morning. For insulin, samples were inverted 5–10 times and centrifuged within 30 minutes of collection. Plasma samples for insulin were stored at −80°C until assays were conducted.

*Blood sample assays.* CRP was assayed using an immunoturbidimetric method with the Architect Ci4100 analyzer latex immunoassay (range of detection = 0.5–75.0 mg/L). Insulin resistance was estimated using the Homeostatic Model Assessment for Insulin Resistance (HOMA-IR) via the following formula: (fasting glucose mg/dL* fasting insulin *µ*U/mL)/405 (Matthews *et al*., 1985). Glucose was assayed using the Architect Ci4100 analyzer (Abbott Laboratory, Chicago IL) via hexokinase/G-6-PDH using the Abbott glucose reagent (range of detection = 5.0–800.0 mg/dL). Insulin was measured as part of the Bio-Plex Pro Human Diabetes Panel (Bio-Rad Laboratories, Hercules, CA, USA) with a detection range of 250–32 000 pg/mL. Insulin levels were calculated as a mean concentration of two duplicate samples. HDL cholesterol was quantified via accelerator-selective detergent on the Architect Ci4100 using the Abbott HDL Ultra reagent (range of detection = 5.0–117.0 mg/dL). Triglyceride level was assayed via glycerol phosphate oxidase using the Architect Ci4100 analyzer (Abbott Laboratory, Chicago IL) with the Abbott triglyceride reagent (range of detection = 5.0–1226.0 mg/dL).

#### Childhood trauma

Childhood trauma was assessed with the 28-item version of the Childhood Trauma Questionnaire (CTQ), which measures experiences of physical/sexual/emotional abuse and physical/emotional neglect in childhood or adolescence using a Likert-style scale ranging 1 (‘never true’) to 5 (‘very often true’) (Bernstein *et al*., 2003). A sum score was calculated for the 28 items (*ω* = 0.97). All participants, regardless of study group, were administered the CTQ to capture the dimensional range of severity in childhood trauma across participants with and without early life stress.

#### Mental health symptoms

Depressive symptoms were measured using the Inventory of Depressive Symptomatology Self Report (IDS-SR) (Rush *et al*., 1986), and was coded as a sum score of the 28 items assessing past-week depressive symptoms on a scale ranging 0 (not present) to 3 (severe) (*ω* = 0.93). Anxious symptoms were measured using the Beck Anxiety Inventory (BAI) (Beck *et al*., 1988). A sum score was created from the 21 items assessing past-week anxious symptoms on a Likert-style scale ranging 0 (‘not at all’) to 3 (‘severely, it bothered me a lot’) (*ω* = 0.93). Posttraumatic stress symptoms were measured with the PTSD Checklist for DSM-5 (PCL-5) (Blevins *et al*., 2015), and was coded as a sum score of the 20 items assessing past-month posttraumatic stress symptoms on a Likert-style scale ranging 0 (‘not at all’) to 4 (‘very often’) (*ω* = 0.96).

#### Health behaviors

Physical activity was measured with the General Practice Physical Activity Questionnaire (GPPAQ) (Department of Health, 2009). Following guidelines from the United Kingdom Department of Health (2009), physical activity was coded as a four-level Physical Activity Index variable based on participants’ responses to 10 items assessing physical activity required by their job and past-week activities outside of work (1 = ‘inactive’, 2 = ‘moderately inactive’, 3 = ‘moderately active’, 4 = ‘active’). Diet quality was approximated using the free, web-based Automated Self-Administered 24-hour (ASA24®) Dietary assessment instrument, which measures past 24-hour food consumption. We coded diet quality as a Healthy Eating Index variable calculated according to Marquez *et al*. (2021) using data from two ASA24 administrations at study visits 2 and 3; this variable ranges 0–100 and estimates overall past 24-hour diet quality across 13 dietary categories based on the 2015–2020 Dietary Guidelines for Americans (DeSalvo *et al*., 2016).

### Analytic plan

We used a network analysis framework to examine the structure of cross-sectional associations between childhood trauma, health behaviors that influence both immunometabolic and psychiatric health (physical activity and diet quality), the immunometabolic biomarkers (CRP, insulin resistance, HDL cholesterol, and triglycerides), and mental health symptoms (depressive, anxious, and post-traumatic stress symptoms). Variables with outliers were Winsorized at the 5th and 95th percentiles, and all variables were standardized (i.e., *z*-scored). Additionally, we adjusted for participant age, sex assigned at birth, body mass index, and study group (denoting the presence or absence of moderate–severe early life stress) by regressing these effects out of each variable prior to analysis.

Because recruitment was stratified by moderate–severe early life stress status, we accounted for study group to ensure that associations between variables were not confounded or obscured by sampling design. Despite adjusting for study group, analyses included childhood trauma (assessed dimensionally with the CTQ) given extensive prior research linking early trauma exposure to the other study variables. The CTQ captures variability in childhood trauma beyond the CECA-defined binary moderate–severe early life stress status. Although participants in the early life stress group had higher CTQ scores on average than the controls (Supplemental Table S1), CTQ scores varied within both groups. All participants in the early life stress group (*n* = 118) and 57.3% of controls (*n* = 47 out of 82) endorsed at least one CTQ item. Thus, childhood trauma exposure was present in a substantial proportion of the control group, despite not meeting the binary threshold for moderate–severe early life stress. To ensure that adjusting for study group did not meaningfully alter relations between childhood trauma and the other study variables, we conducted a sensitivity analysis comparing the network structure with versus without adjusting for study group.

We used the *psychonetrics* package v0.13 to estimate a Gaussian graphical model (GGM), a type of network model for multivariate continuous data (Epskamp *et al*., 2018; Isvoranu & Epskamp, 2023; Epskamp, 2024). In the GGM, network *nodes* represent the study variables, and *edges* represent undirected partial correlations between each pair of variables after adjusting for other effects in the model. The GGM was estimated using the nlmib optimizer, and missing data were handled with Full Information Maximum Likelihood (see Table 2 for missingness rates) (Epskamp, 2020; Isvoranu & Epskamp, 2023; Kline, 2023). We used a three-step recursive model search procedure to identify the best-performing GGM and to avoid spurious edges: first, pruning fixed edges with *p* > 0.05 to 0 before re-estimating the model; second, remaining edges were iteratively re-added until the optimal (lowest) BIC was obtained; third, a second pruning step again fixed edges *p* > 0.05 to 0 (Blanken *et al*., 2022). After selecting the best-performing model, we assessed edge stability using a case-drop bootstrap resampling procedure, in which each model was refit 1000 times using random subsamples of *n* = 160 participants (80% of the full sample) without replacement (Epskamp, 2020). Edges included in a higher proportion of the bootstrapped models were considered more stable. Lastly, we assessed the relative importance of nodes in the network structure by inspecting centrality indices, including node strength, closeness, and betweenness (Hevey, 2018; Deserno *et al*., 2022). The network structure was visualized using *qgraph* (Epskamp *et al*., 2012).

**Table 2.** Descriptive statistics, missingness, and bivariate correlations for the 10 modeled variables (*N* = 200)

			Bivariate correlations								
	*M* ± *SD*	*n* Missing (%)	1.	2.	3.	4.	5.	6.	7.	8.	9.
1. C-reactive protein	2.69 ± 3.17	1 (0.50%)	–	–	–	–	–	–	–	–	–
2. Insulin resistance	12.36 ± 9.78	5 (2.50%)	0.55	–	–	–	–	–	–	–	–
3. HDL cholesterol	54.56 ± 13.93	6 (3.00%)	−0.15	−0.42	–	–	–	–	–	–	–
4. Triglycerides	89.06 ± 0.76	6 (3.00%)	0.20	0.40	−0.28	–	–	–	–	–	–
5. Childhood trauma	49.74 ± 24.21	0 (0.00%)	0.16	0.29	−0.18	0.04	–	–	–	–	–
6. Depressive symptoms	13.01 ± 10.64	0 (0.00%)	0.06	0.19	−0.15	0.02	0.60	–	–	–	–
7. Anxious symptoms	4.88 ± 6.05	0 (0.00%)	0.02	0.16	−0.10	0.00	0.57	0.72	–	–	–
8. Posttraumatic stress symptoms	13.10 ± 14.80	20 (10.00%)	0.11	0.15	−0.11	0.02	0.66	0.70	0.61	–	–
9. Physical activity	2.99 ± 1.15	17 (8.50%)	−0.11	−0.26	0.02	−0.07	−0.08	−0.18	−0.08	−0.07	–
10. Diet quality	54.19 ± 12.54	72 (36.00%)	−0.14	−0.14	0.16	−0.14	−0.11	−0.19	−0.18	−0.07	0.10

*Note*: Coefficients represent descriptive statistics and bivariate correlations prior to standardizing variables and adjusting for the effects of age, sex assigned at birth, body mass index, and the presence or absence of early life stress (i.e., study group for the overall LIFE study). Variables approximated normal distributions after adjusting for these covariates. Variables with outliers were Winsorized at the 5th and 95th percentiles.

Regarding statistical power, we conducted an *a priori* power analysis via Monte Carlo simulations with the *powerly* package v1.8.6, using 500 replications for each of 100 candidate sample sizes ranging *n* = 100–300. Results indicated that a sample size of *N* = 152 yields 80% power to observe a sensitivity of ≥0.80 in a GGM with 10 nodes and a network density of 0.25 (i.e., we conservatively estimated that 25% of total possible edges would be retained in the final model) (Constantin *et al*., 2023). Thus, our sample size of *N* = 200 was considered sufficient for the proposed analysis. The *R* code for our analyses is provided in the Appendix, and the data that support the findings of this study are available from the senior author upon reasonable request.

## Results

**Figure 1.**
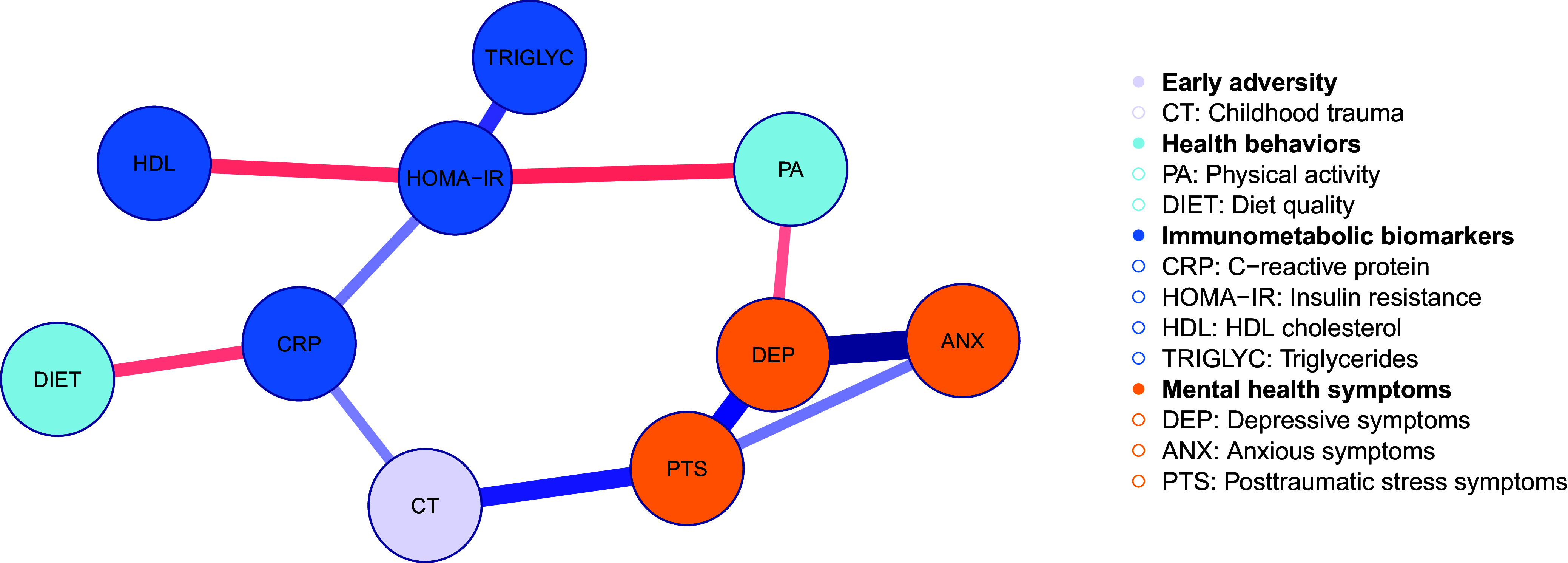
Visualized network structure for the pruned estimated Gaussian graphical model. Lines represent undirected partial correlations (edges) between each pair of variables (*N* = 200). Edge thickness denotes effect size, with thicker, darker lines indicating stronger effects. Edge color represents effect direction (red = negative, blue = positive). Edges not shown were pruned during model selection. Corresponding numerical estimates are shown in Table 2.

**Figure 2.**
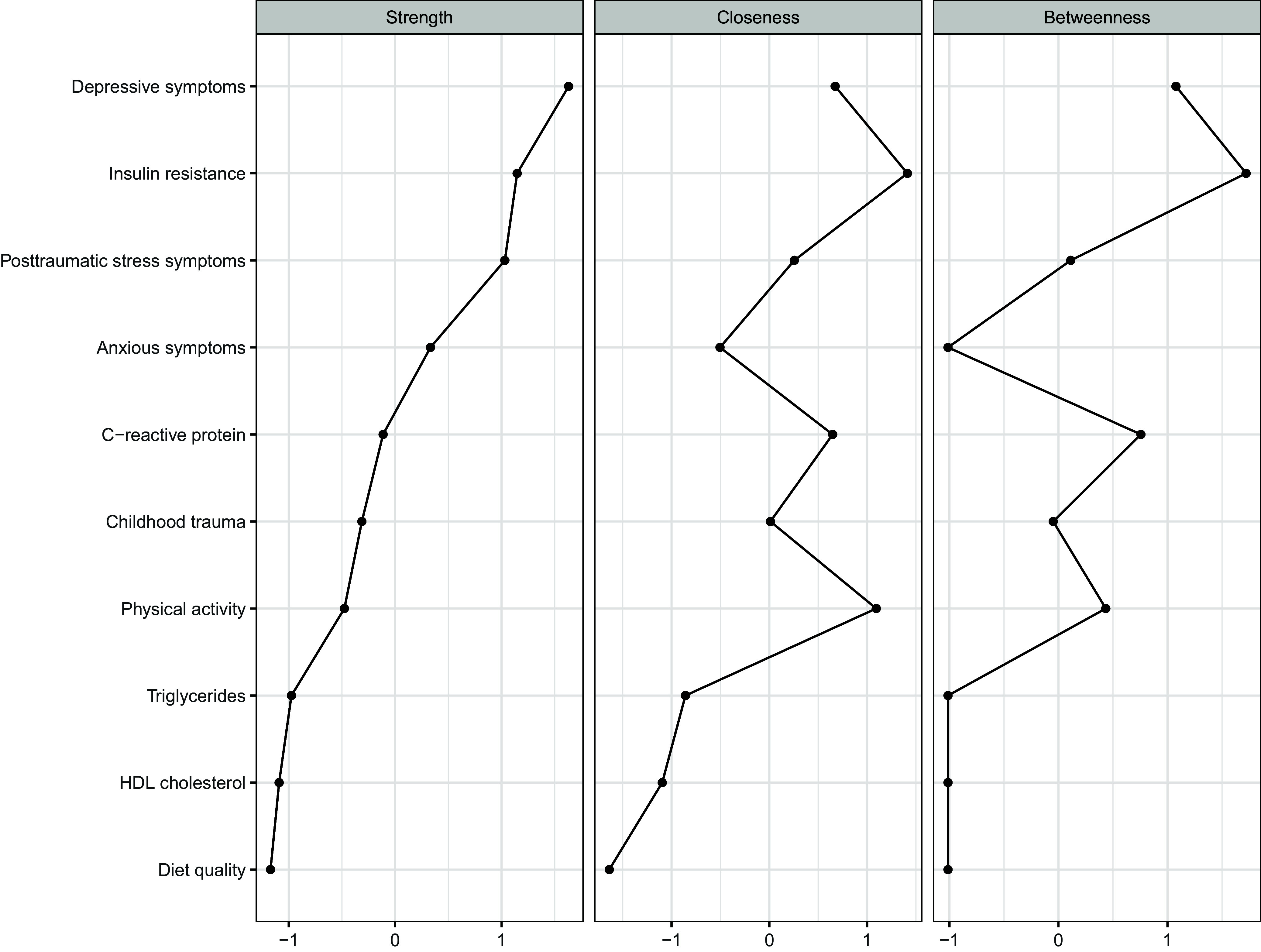
Node centrality indices for the pruned estimated Gaussian graphical model (*N* = 200). Centrality values are shown in the metric of *z*-scores. Strength reflects the sum of absolute edge weights directly connected to a node. Closeness reflects how close (in geodesic distance) a node is to other nodes in the network. Betweenness reflects how often a node is on the shortest path between other nodes (Hevey, 2018; Deserno *et al*., 2022).

Descriptive statistics, missingness, and bivariate correlations for the 10 modeled variables (i.e., network nodes) are shown in Table 2. To provide further context on the sample, descriptive statistics and missingness for the modeled variables across the two LIFE study groups (early life stress group versus controls) are shown in Supplemental Table S1. The pruned network structure for the GGM is visualized in Figure 1, and corresponding model estimates and bootstrap inclusion probabilities for each edge are shown in Table 3. Centrality indices for each node are presented in Figure 2. Interpretation primarily focused on the network structure, as our main analytic goal was to understand patterns of conditional dependencies among the variables. In the pruned estimated GGM, physical activity and childhood trauma appeared to provide potential links between clusters of the immunometabolic biomarkers and mental health symptoms (Figure 1, Table 3). Positive pairwise associations were observed between all three mental health symptom nodes (depressive, anxious, and posttraumatic stress symptoms), suggesting comorbidity in these symptoms within the sample. Among the immunometabolic biomarkers, insulin resistance directly associated with lower HDL cholesterol, higher triglyceride levels, and higher CRP. Physical activity associated with lower insulin resistance and fewer past-week depressive symptoms. Thus, higher physical activity may be protective against both insulin resistance and depressive symptoms, and individuals with lower depressive symptoms and lower insulin resistance may engage in less physical activity. Childhood trauma positively associated with both CRP and past month posttraumatic stress symptoms, suggesting childhood trauma increased risk for both posttraumatic stress symptoms and higher inflammation in adulthood. Lastly, CRP was associated with lower diet quality.

**Table 3.** Estimated undirected partial correlations and bootstrap inclusion probabilities for the pruned Guassian graphical model (GGM) (*N* = 200)

	1.	2.	3.	4.	5.	6.	7.	8.	9.	10.
1. C-reactive protein		**0.79**	0.00	0.00	**0.77**	0.00	0.01	0.00	0.00	**0.74**
2. Insulin resistance	0.17 ± 0.06**		**0.97**	**0.99**	0.01	0.15	0.00	0.01	1.00	0.00
3. HDL cholesterol	–	−0.23 ± 0.06***		0.04	0.00	0.00	0.00	0.00	0.00	0.00
4. Triglycerides	–	0.27 ± 0.06***	–		0.00	0.03	0.00	0.00	0.02	0.00
5. Childhood trauma	0.15 ± 0.06*	–	–	–		0.02	0.02	**0.99**	0.00	0.00
6. Depressive symptoms	–	–	–	–	–		**1.00**	**1.00**	**0.94**	0.00
7. Anxious symptoms	–	–	–	–	–	0.49 ± 0.05***		**0.41**	0.01	0.08
8. Posttraumatic stress symptoms	–	–	–	–	0.31 ± 0.05***	0.39 ± 0.06***	0.17 ± 0.07*		0.02	0.00
9. Physical activity	–	−0.24 ± 0.06***	–	–	–	−0.17 ± 0.05***	–	–		0.07
10. Diet quality	−0.21 ± 0.08*	–	–	–	–	–	–	–	–	

*Note*: The lower triangle shows partial correlations (edges) and accompanying standard errors retained in the full sample pruned network model (visualized in Figure 1.). Edges with *p* > 0.05 were fixed to zero during model estimation. The upper triangle shows the proportion of times each edge was retained in the network structure across the 1000 case-drop bootstrapped models using random subsamples of *n* = 160. Bolded values represent edges that were retained in the original full-sample model. HDL = high-density lipoprotein. *p < 0.05, **p < 0.01, ***p < 0.001.

Except for one edge (the positive association between anxious and posttraumatic stress symptoms), bootstrap inclusion probabilities were all ≥0.74 for edges retained in the full-sample model, suggesting good stability of these effects (Table 3). Further, the generally low bootstrap inclusion probabilities for edges pruned out of the full-sample model (≤0.15) increased confidence in model specificity. Insulin resistance, depressive symptoms, and posttraumatic stress symptoms demonstrated the highest strength centrality, indicating these variables had the strongest direct associations with other nodes in the network (Figure 2) (Hevey, 2018; Deserno *et al*., 2022). Insulin resistance, depressive symptoms, and physical activity showed the highest betweenness centrality, while insulin resistance, depressive symptoms, and CRP exhibited the highest closeness centrality. These patterns for betweenness and closeness centrality suggest that insulin resistance, depressive symptoms, CRP, and physical activity may indirectly influence – or be influenced by – other nodes in the network (Hevey, 2018; Deserno *et al*., 2022). Results for the sensitivity analysis examining the network structure without adjustment for study group are presented in Supplemental Table S2 and Supplemental Figure S2. This network structure was nearly identical to the original model, except for one additional edge between childhood trauma and depressive symptoms when study group was not adjusted for. Overall, adjustment for study group did not meaningfully alter the network structure.

## Discussion

This study used network analysis to examine interdependencies between early life stress (childhood trauma), health behaviors (diet quality, physical activity), immunometabolic biomarkers reflecting metabolic functioning (insulin resistance, HDL cholesterol, triglycerides) and inflammation (CRP), and three domains of mental health symptoms (depressive, anxious, and posttraumatic stress symptoms) in a community sample of medically healthy adults with and without early life stress. The cross-sectional network structure suggested the positive association between higher levels of CRP and childhood trauma was associated with increased risk for both metabolic impairments and mental health symptoms in adulthood. Further, higher physical activity was associated with lower insulin resistance and fewer depressive symptoms, and better diet quality was associated with lower CRP levels. While the cross-sectional nature of this study precludes interpreting causal or temporal relations between variables, results provide support for the potential roles of inflammation and childhood trauma in metabolic and psychiatric comorbidities in adulthood, as well as the potential protective effects of modifiable health behaviors on these processes. Given that the sample was comprised of medically healthy adults without acute or chronic conditions, the observed associations between mental health symptoms and immunometabolic biomarkers likely reflect subclinical impairments indicative of early-stage metabolic dysfunction, thereby highlighting potential opportunities for early intervention.

The network structure identified separate clusters of metabolic and mental health variables. The three mental health symptom domains were all interrelated, particularly the paths between depressive and posttraumatic stress symptoms and between depressive and anxious symptoms. This aligns with robust literature on the frequent comorbidity of mental health symptom dimensions (Eaton *et al*., 2023) and suggests that interventions to improve one symptom domain may have positive effects on the others. Among the biomarkers, insulin resistance stood out as a salient feature of the network system. Insulin resistance had high centrality parameters and demonstrated direct associations with the other three biomarkers, as well as potential indirect paths to depressive symptoms via physical activity and to childhood trauma via CRP. Prior research suggests non-diabetic psychiatric patients with impaired glycemic regulation (e.g., elevated insulin resistance, prediabetes) have more severe psychopathology over time and poorer response to treatment compared to matched normoglycemic psychiatric controls (Calkin *et al*., 2015; Mansur *et al*., 2016; Steardo *et al*., 2019; Miola *et al*., 2023). Thus, glycemic regulation may be a promising focus of future research on etiology and interventions for mental health conditions. Correspondingly, it is well-established that behavioral changes to increase physical activity and improve diet can improve insulin resistance and blood lipid profiles, reduce inflammation, and alleviate mental health symptoms (Giugliano *et al*., 2006; Vos *et al*., 2011; Margină *et al*., 2020; Burrows *et al*., 2022; Singh *et al*., 2023). In the network structure, physical activity had direct negative associations with insulin resistance and depressive symptoms, while better diet quality was associated with lower inflammation (i.e., lower CRP). Hence, results support previous literature highlighting that health behaviors can have important protective effects against immunometabolic and mental health problems and thus represent potential targets for understanding risk and preventing and/or intervening on comorbid conditions.

Results also support previous literature suggesting a potential inflammatory pathway through which early life stress impacts metabolic and psychiatric health in adulthood. In the network model, CRP directly associated with insulin resistance and childhood trauma and demonstrated a potential indirect path to the cluster of mental health symptom variables via childhood trauma. While the cross-sectional design of the current study prevents establishing temporality between variables, prior research suggests that childhood trauma may have long-lasting impacts on immune functioning, resulting in chronic elevated inflammatory activity in adulthood (Den Noortgate *et al*., 2025). Chronic inflammation can subsequently have self-reinforcing associations with metabolic problems, increasing vulnerability to insulin resistance and unhealthy blood lipid profiles (Gómez-Ilescas & Silveira, 2025a). Chronic inflammation, particularly during critical periods of development, may also impact neurotransmitter pathways, neural circuits, and stress-response systems, thereby increasing predisposition for psychiatric concerns across the lifespan (Shin & Kim, 2023). Thus, continued research to understand inflammation in the context of childhood trauma and health outcomes in adulthood is an important direction for future work. Interventions that can reduce inflammation and improve metabolic health (e.g., physical activity and diet interventions) may be particularly promising among individuals with early life stress, who experience greater vulnerability poorer health outcomes.

Some limitations of the current study highlight important directions for future research. First, our sample was 68% White and 83% non-Hispanic, yet racial and ethnic minority groups experience a disproportionate burden of psychiatric and metabolic conditions (Bailey *et al*., 2017; Barksdale *et al*., 2022). Replicating our results in more diverse samples, including those with identity-related discrimination and stress, is important for clarifying the generalizability of our findings. Second, statistical power and effect stability in network models are limited by the ratio of variables to sample size (Constantin *et al*., 2023). While our analyses included multiple key metabolic biomarkers, psychiatric symptom domains, and relevant covariates, our sample of *N* = 200 limited the number of variables we could consider (10 variables, to retain sufficient power for the bootstrapping procedure), and additional factors likely impact these processes. Future studies should replicate and extend our results in larger samples that consider socioenvironmental contexts that influence both mental and physical health (e.g., socioeconomic status, family history) and additional biomarkers of related physiological systems (e.g., mitochondrial processes, plasminogen activator inhibitor-1) (Daniels *et al*., 2020; Kudinova *et al*., 2024). Third, the cross-sectional design prevented interpreting causal or temporal effects, and some variables reflected processes that operate on different timescales. For example, blood-based CRP has a half-life of approximately 19 hours (Vigushin *et al*., 1993), while insulin resistance and blood lipids reflect more stable physiological states across weeks or months (Poon *et al*., 2018). Employing longitudinal approaches to clarify the timescales and causal directions of observed associations and how these vary across people could inform precision intervention approaches.

### Conclusions

Despite the abovementioned limitations, the current study provides valuable information on interdependencies between childhood trauma, health behaviors (diet quality, physical activity), immunometabolic biomarkers (insulin resistance, HDL cholesterol, triglycerides, CRP), and multiple domains of mental health symptoms (depressive, anxious, and posttraumatic stress symptoms) in adults. Results from the cross-sectional network model suggests that psychiatric and metabolic comorbidities may stem from complex interrelations among metabolic functioning, immune response, health behaviors, and mental health. Findings from this work highlight the potentially intermediary roles of childhood trauma and immune function between mental health and metabolic functioning. Results also underscore potential avenues for future interventions focused on reducing inflammation and improving health behaviors to address psychiatric and metabolic comorbidities. Continued efforts in precision medicine that leverage the intersection of individual variability in lifestyle, biomarkers, and medical history promise to shift the paradigm closer to the unique needs of each individual.

## Supporting information

10.1017/neu.2026.10069.sm001 Wallace et al. supplementary material Wallace et al. supplementary material

## Data Availability

The data that support the findings of this study are available from the senior author upon reasonable request.

## Supplementary material

The supplementary material for this article can be found at https://doi.org/10.1017/neu.2026.10069.
