# Supplementary material for: Childhood trauma and physical activity link immunometabolic biomarkers and psychiatric symptoms in medically healthy adults
Source: Acta Neuropsychiatr. 2026 Mar 25;38:e28. doi: 10.1017/neu.2026.10069 (PMC13110904; doi:10.1017/neu.2026.10069)
Supplement: Wallace et al. supplementary material [file S0924270826100696sup001.docx]

**Appendix**

**R Code for Gaussian Graphical Model (GGM) Estimation and Evaluation**

### Power analysis for GGM ###

ggmpower <- powerly(range_lower = 100,

range_upper = 300,

samples = 100,

replications = 500,

measure = "sen",

measure_value = 0.8,

statistic = "power",

statistic_value = 0.8,

boots = 10000,

model = "ggm",

nodes = 10,

density = 0.25)

summary(ggmpower)

plot(ggmpower, step=1)

plot(ggmpower, step=2)

plot(ggmpower, step=3)

### Estimate GGM ###

ggm <- ggm(vars, estimator = "FIML", optimizer = "nlminb", storedata=TRUE) %>%

runmodel %>%

prune(alpha = 0.05) %>%

stepup(criterion = "bic") %>%

prune(alpha = 0.05)

### Assess GGM parameters ###

ggm %>% print

ggm %>% parameters

omega <- ggm %>% getmatrix("omega")

### Bootstrap GGM ###

bootstraps <- pbmclapply(1:1000, function(x) {

bootstrapped_model <-

ggm(

vars [sample(1:nrow(vars), 160,

replace = FALSE), ],

estimator = "FIML",

optimizer = "nlminb",

storedata=TRUE) %>%

runmodel %>%

prune(alpha = 0.05) %>%

stepup(criterion = "bic") %>%

prune(alpha = 0.05)

return(bootstrapped_model)

},

mc.cores = detectCores() - 2)

### Visualize GGM network structure ###

labels <- c("CRP", "HOMA-IR", "HDL", "TRIGLYC", "CT", "DEP", "ANX", "PTS", "PA", "DIET")

groups <- c("Immunometabolic biomarkers", "Immunometabolic biomarkers", "Immunometabolic biomarkers", "Immunometabolic biomarkers", "Early adversity", "Mental health symptoms", "Mental health symptoms", "Mental health symptoms", "Health behaviors", "Health behaviors")

names <- c("C-reactive protein", "Insulin resistance", "HDL cholesterol", "Triglycerides", "Childhood trauma", "Depressive symptoms", "Anxious symptoms", "Posttraumatic stress symptoms", “Physical activity", "Diet quality")

qgraph(omega,

labels = labels,

label.cex = 1,

label.prop = 0.8,

label.scale = F,

label.scale.equal = T,

edge.labels = F,

groups = groups,

nodeNames = names,

layout = "spring",

theme = "Borkulo",

legend = T,

legend.cex = 0.6,

legend.mode = 'style2',

layoutOffset = c(-.17,0),

layoutScale = c(1, 0.65),

repulsion = 0.93,

color = c("lavender","lightblue2","dodgerblue2","orange1"),

width = 9.2,

height = 7,

filetype="pdf",

filename = "Figure1.pdf")

### Visualize GGM centrality indices ###

pdf("FigureS1.pdf",width=9.2,height=7)

full_centrality <- centralityPlot(omega,

scale = "z-scores",

labels = names,

print=TRUE,

theme_bw = T,

orderBy = "Strength",

include = c("Strength", "Closeness", "Betweenness"))

dev.off()

**Supplemental Tables**

| Supplemental Table S1. Descriptive statistics and missingness for the 10 modeled variables across the two LIFE study groups. | | | | |
| --- | --- | --- | --- | --- |
|  | Early life stress group (*n* = 118) | | Control group (*n* = 82) | |
|  | *M* ± *SD* | *n* Missing (%) | *M* ± *SD* | *n* Missing (%) |
| 1. C-reactive protein | 2.95 ± 3.30 | 1 (0.85%) | 2.32 ± 2.94 | 0 (0.00%) |
| 2. Insulin resistance | 14.04 ± 10.45 | 3 (2.54%) | 9.95 ± 8.20 | 2 (2.44%) |
| 3. HDL cholesterol | 52.60 ± 13.87 | 3 (2.54%) | 57.41 ± 13.60 | 3 (3.36%) |
| 4. Triglycerides | 90.95 ± 41.32 | 3 (2.54%) | 86.32 ± 40.03 | 3 (3.36%) |
| 5. Childhood trauma | 65.42 ± 19.65 | 0 (0.00%) | 27.20 ± 3.11 | 0 (0.00%) |
| 6. Depressive symptoms | 17.78 ± 11.10 | 0 (0.00%) | 6.15 ± 4.41 | 0 (0.00%) |
| 7. Anxious symptoms | 6.98 ± 6.71 | 0 (0.00%) | 1.85 ± 3.05 | 0 (0.00%) |
| 8. Posttraumatic stress symptoms | 19.65 ± 15.73 | 14 (11.86%) | 4.13 ± 6.47 | 6 (7.32%) |
| 9. Physical activity | 2.92 ± 1.19 | 12 (10.17%) | 3.10 ± 1.08 | 5 (6.10%) |
| 10. Diet quality | 52.04 ± 13.23 | 45 (38.14%) | 57.04 ± 11.04 | 27 (32.93%) |
| *Note*: The two study groups were combined in the Gaussian graphical models. Sensitivity analyses indicated that adjusting for study group did not meaningfully impact network model results (see Supplemental Table S2 and Figure S1). | | | | |

| Supplemental Table S2. Estimated undirected partial correlations and bootstrap inclusion probabilities for the pruned Gaussian graphical model (GGM) without adjusting for study group (*N*=200). | | | | | | | | | |
| --- | --- | --- | --- | --- | --- | --- | --- | --- | --- |
|  | 1. | 2. | 3. | 4. | 5. | 6. | 7. | 8. | 9. |
| 1. C-reactive protein |  |  |  |  |  |  |  |  |  |
| 2. Insulin resistance | .16 ± .06** |  |  |  |  |  |  |  |  |
| 3. HDL cholesterol | – | -.23 ± .06*** |  |  |  |  |  |  |  |
| 4. Triglycerides | – | .26 ± .06*** | – |  |  |  |  |  |  |
| 5. Childhood trauma | .13 ± .05* | – | – | – |  |  |  |  |  |
| 6. Depressive symptoms | – | – | – | – | .20 ± .06*** |  |  |  |  |
| 7. Anxious symptoms | – | – | – | – | – | .53 ± .05*** |  |  |  |
| 8. Posttraumatic stress symptoms | – | – | – | – | .35 ± .06*** | .35 ± .06*** | .20 ± .07** |  |  |
| 9. Physical activity | – | -.25 ± .06*** | – | – | – | -.14 ± .04*** | – | – |  |
| 10. Diet quality | -.22 ± .08** | – | – | – | – | – | – | – | – |
| *Note*: This table shows results for the sensitivity analysis to assess if the network structure changed when study group (i.e., the presence or absence of moderate-severe early life stress) was not globally residualized out of analytic variables. The lower triangle shows partial correlations (edges) and accompanying standard errors retained in the pruned network model (visualized in Figure 1). Edges with *p*>.05 were fixed to zero during model estimation. HDL = high-density lipoprotein. **p*<0.05, ***p*<0.01, ****p*<0.001 | | | | | | | | | |

**Supplemental Figures**

****Supplemental Figure S1. Visualized network structure for the pruned estimated Gaussian graphical model without adjusting for study group (*N*=200). This figure shows results for the sensitivity analysis to assess if the network structure changed when study group (i.e., the presence or absence of moderate-severe early life stress) was not globally residualized out of analytic variables. Age, sex, and BMI were still adjusted for in the presented model. Lines represent undirected partial correlations (edges) between each pair of variables. Edge thickness denotes effect size, with thicker, darker lines indicating stronger effects. Edge color represents effect direction (red = negative, blue = positive). Edges not shown were pruned during model selection. Corresponding numerical estimates are shown in Supplemental Table S2.
